# Network Security Situation Prediction Based on Optimized Clock-Cycle Recurrent Neural Network for Sensor-Enabled Networks

**DOI:** 10.3390/s23136087

**Published:** 2023-07-01

**Authors:** Xiuli Du, Xiaohui Ding, Fan Tao

**Affiliations:** Communication and Network Laboratory, Dalian University, Dalian 116622, China; dingxiaohui@s.dlu.edu.cn (X.D.); tfxzhmu520@163.com (F.T.)

**Keywords:** network security, situation prediction, Grey Wolf Optimization (GWO), Clockwork Recurrent Neural Networks (CW-RNN)

## Abstract

We propose an optimized Clockwork Recurrent Neural Network (CW-RNN) based approach to address temporal dynamics and nonlinearity in network security situations, improving prediction accuracy and real-time performance. By leveraging the clock-cycle RNN, we enable the model to capture both short-term and long-term temporal features of network security situations. Additionally, we utilize the Grey Wolf Optimization (GWO) algorithm to optimize the hyperparameters of the network, thus constructing an enhanced network security situation prediction model. The introduction of a clock-cycle for hidden units allows the model to learn short-term information from high-frequency update modules while retaining long-term memory from low-frequency update modules, thereby enhancing the model’s ability to capture data patterns. Experimental results demonstrate that the optimized clock-cycle RNN outperforms other network models in extracting the temporal and nonlinear features of network security situations, leading to improved prediction accuracy. Furthermore, our approach has low time complexity and excellent real-time performance, ideal for monitoring large-scale network traffic in sensor networks.

## 1. Introduction

The rapid development of cyberspace has brought about both convenience and challenges in terms of network security. Traditional network defense techniques relying on log analysis from a single detection device may result in a biased security situation analysis due to limitations in data acquisition channels and uncertainties in detection devices. To address these issues, the modern network security needs to shift from passive defense to active perception. Network Security Situation Awareness (NSSA) [1], particularly situation prediction, plays a crucial role in improving the performance of traditional security defense tools [2]. Network security situation prediction has gained continuous attention from researchers globally [3].

In recent practical applications, sensor networks have been increasingly utilized to perceive security events, intrusion behavior, and abnormal activities within networks, thus enhancing the accuracy and efficiency of network security prediction [4]. However, traditional perception models suffer from limitations such as inconsistent function construction [5], reliance on expert knowledge [6], subjectivity [6], poor scalability [5], and low performance [6]. On the other hand, neural networks have also shown promising results when applied to network-related areas, such as network traffic anomaly detection [7] and network traffic prediction [8].

In this paper, we propose an optimized Grey Wolf Optimized Clockwork Recurrent Neural Network (CW-RNN) model to address the low accuracy of existing network security situation prediction methods. By analyzing network traffic attack data collected by sensor networks using the optimized neural network model, our research aims to improve the accuracy and real-time performance of network security predictions, effectively addressing network security challenges.

### 1.1. Main Contributions

Our study proposes a Grey Wolf Optimization (GWO) algorithm optimized Clockwork Recurrent Neural Network (CW-RNN) network security situation prediction model. Our work makes the following contributions:

- Capturing the temporal characteristics of network security postures and optimization: CW-RNN combines the memory capabilities of Recurrent Neural Networks with a periodic update mechanism. This enables the model to better capture the temporal characteristics of network security postures, improving computational efficiency and real-time performance. Additionally, we employ the GWO algorithm to optimize the CW-RNN model’s hyperparameters, efficiently searching the hyperparameter space for an optimal combination. This optimization enhances the model’s performance and robustness in network security posture prediction.
- Comparative experiments with other algorithms: To demonstrate the superiority of the GWO algorithm, we conduct comparative experiments with other commonly used optimization algorithms such as Genetic Algorithm and Particle Swarm Optimization. By comparing the performance of different algorithms within the same hyperparameter search space, we validate the outstanding effectiveness of the GWO algorithm in optimizing the CW-RNN model.
- Model comparison experiments: To evaluate the performance of our proposed CW-RNN model, we conduct model comparison experiments. We select several widely used models, such as LSTM, GRU, and Transformer as benchmark models. By training and testing these models on the same dataset and comparing their performance metrics, we can demonstrate the superiority of the CW-RNN in network security posture prediction tasks.

### 1.2. Structure of the Paper

The rest of the paper is organized as follows. First, we present the literature review in Section 2. Then, we introduce the related model and algorithms in Section 3. We describe the design of the optimized clock-period Recurrent Neural Network in Section 4. In Section 5, we analyze and compare the optimized clock-period Recurrent Neural Network proposed in this paper. Finally, some conclusions are given in Section 6.

## 2. Related Works

Probabilistic reasoning models such as attack graph models [9] and Bayesian models [10] were used in early studies of network security situation prediction models. They predicted the evolution trends of individual or compound attack events [11]. However, due to the complex nonlinear characteristics of situation data, these models have certain limitations in practical applications. To overcome these issues, many researchers have turned to neural network algorithms for studying network security situation prediction [12].

When the dataset of situation data is small, researchers tend to use Support Vector Machines (SVM) [13]. Sun Weixi proposed an improved particle swarm algorithm to optimize SVM for network security situation prediction, which improved the prediction accuracy compared to linear prediction methods [14].

On the other hand, Hu et al. proposed a MapReduce-based SVM state prediction algorithm, which solved the problem of long training time for SVM under a large number of samples and improved the prediction accuracy while reducing training time [13]. However, with the increase in network security data, the performance of traditional prediction models gradually declines.

Zhang et al. proposed using BP neural networks to predict network security situations, utilizing the guided-head optimization algorithm to search for optimal weights and introducing the simulated annealing algorithm to enhance the global search capability of the algorithm [15]. However, BP neural networks face difficulties in handling temporal data due to the lack of an internal memory mechanism, which leads to performance degradation in network intrusion detection tasks.

Li et al. proposed a model based on Recursive Neural Networks (RNN) and its variants, which effectively improved the accuracy of feature representation through recursive stacking network structures and feature separation methods based on the temporal characteristics of intrusion data [16]. However, this approach still faces the problem of overfitting. On the other hand, a study [17] compared several network security situation prediction methods, and the results showed that a radial basis function neural network optimized by a particle swarm had good performance. However, this method used a small number of data samples, which may limit the model’s generalization ability and representativeness of sample distribution, thus restricting its application and performance in real network security environments.

Another related work [18] proposed a network security situation assessment method based on a dual attention mechanism and HHO-ResNeXt, which combined ResNeXt with the efficient channel attention (ECA) module and the contextual transformer (COT) block to enhance the convolutional neural network. The proposed method also used a Harris Hawks optimization (HHO) algorithm to select the optimal hyperparameters of the model. The experimental results showed that this method had high accuracy and low false positive rate in assessing a network security situation, and outperformed other methods such as k-means clustering and support vector machine (SVM). However, this method did not consider the temporal features of a network security situation, and only used a single ResNeXt model for assessment, which may limit the model’s ability to handle complex and dynamic network data.

Another related work [19] proposed a network security situation prediction method based on Attention-CNN-BiGRU, which combined a convolutional neural network (CNN) and a bidirectional gated recurrent unit (BiGRU) to extract and predict the network security situation features. The proposed method also used an attention mechanism to assign different weights to different feature maps according to their importance for prediction and used a particle swarm optimization (PSO) algorithm to optimize the hyperparameters of the model. The experimental results showed that this method had high accuracy and low error in predicting the network security situation, and outperformed other methods such as LSTM, GRU, and TCN. However, this method did not consider the spatial features of a network security situation, and only used a single CNN-BiGRU model for prediction, which may limit the model’s ability to handle complex and dynamic network data.

In order to fully extract the temporal and nonlinear characteristics of network intrusion detection data, many scholars have started using Recurrent Neural Networks (RNN) and their variants. The Zhu Jiang team has made outstanding contributions to security situation prediction. Starting from the temporal features of network security situations, they proposed an improved Long Short-Term Memory (LSTM) situation awareness algorithm and accelerated the convergence speed of LSTM through the optimized NAdam algorithm. Experimental results showed that this algorithm had a small mean square error [20]. Subsequently, they added an attention mechanism to the Gated Recurrent Unit (GRU), which automatically allocated weights to input situation data, resulting in a low level of absolute prediction error [21]. However, complex models often require more computational resources and time for training and inference, which reduces the real-time performance of the model, especially in tasks that require real-time response, such as network intrusion detection, particularly when monitoring large-scale network traffic using sensor networks.

Another recent study [22] proposed a network security situation awareness method based on the LSTM-DT model, which combined LSTM with the decision tree (DT) algorithm. The LSTM network was used to predict the attack probability of each network packet, and the DT algorithm was used to identify the attack type. The model also introduced the concept of attack impact to evaluate the network security situation level. The experimental results showed that this method had high accuracy and low error in predicting the network security situation and outperformed several baseline methods. However, this method did not consider the spatial correlation among network packets, which may affect the model’s ability to capture complex attack patterns. Moreover, this method did not take into account the long-term features of the network security situation, and used a fixed time step for LSTM, which may have led to different sensitivities of the model to different frequency data features, affecting the model’s generalization ability.

In addition, Zhao Dongmei proposed a network security situation prediction method based on Transformer, which reduced the difficulty of training required with Transformer by introducing GRU to reduce the dimensionality of sample features. This method showed good performance on two network security datasets [23]. However, the Transformer model still has disadvantages such as a high training cost, the need for a large amount of computational resources, and limited interpretability.

Compared to existing research on network security situation prediction, our optimized Clockwork Recurrent Neural Network model offers the following advantages (Table 1):

## 3. GWO-Optimized CW-RNN-Based Situation Prediction Algorithm

### 3.1. Clockwork RNN

**Figure 1:**
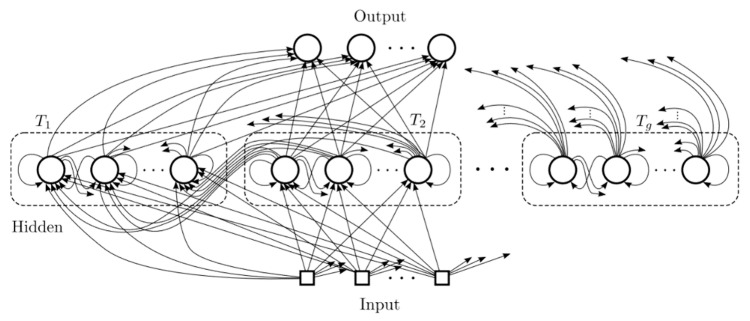
CW-RNN network structure.

The Clockwork RNN (CW-RNN) [24] is a neural network that solves the problem of long-term dependency in time series learning by adjusting the connectivity structure of RNN [25] hidden layers. Its most distinctive feature is the use of a clock-driven parameter updating mechanism. The hidden layers of CW-RNN are divided into independent modules, and each module is assigned a clock that controls the processing of the input [26]. Because the clock period determines the update speed of each module, slower modules are required to connect to faster ones to ensure information transmission. The network structure of CW-RNN is shown in Figure 1, where the square represents input information, the circle represents a neuron, and the arrow represents the direction of connectivity, i.e., the direction of information propagation.

Figure 1 illustrates the hidden layer of CW-RNN, where neurons are divided into g modules, and each module operates with a different running clock $T_{i}\in \left\{T_{1},\ldots ,T_{g}\right\}$. The frequencies, from left to right, represent the descending order of module update rates. Here, the frequency refers to the rate at which the modules are updated, with smaller clock periods indicating faster updates and larger clock periods indicating slower updates. $T_{1}$ corresponds to the shortest period with the fastest module updates, while $T_{g}$ corresponds to the longest period with the slowest module updates. The neurons within each module in the hidden layer are fully connected, while the connections between modules always occur from slower clock modules to faster clock modules. In other words, the modules with longer periods on the right side point to the modules with shorter periods on the left side. This arrangement ensures that the modules with lower update frequencies maintain longer-term memory without being influenced by the modules with higher update frequencies, while the higher-frequency modules incorporate long-term memory information from the lower-frequency modules.

The forward propagation calculation of CW-RNN is similar to that of the standard RNN. The hidden layer output $y_{H}^{\left(t\right)}$ at time t and the final output $y_{O}^{\left(t\right)}$ are calculated using Equations (1) and (2), respectively, in standard RNN:

(1) $$y_{H}^{\left(t\right)}=f_{H}\left(W_{H}\cdot y_{H}^{\left(t-1\right)}+W_{I}\cdot x^{\left(t\right)}+b_{H}\right)$$

(2) $$y_{O}^{\left(t\right)}=f_{O}\left(W_{O}\cdot y_{H}^{\left(t\right)}+b_{O}\right)$$

Here, $W_{H}$, $W_{I}$, and $W_{O}$ are the weight parameters of the hidden layer, input layer, and output layer, respectively. Variable $x^{\left(t\right)}$ represents the input at time t, $y_{H}^{\left(t-1\right)}$ denotes the output of the hidden layer at time t−1, and $f_{H}\left(\right)$, $f_{O}\left(\right)$ represent the non-linear activation functions of the hidden layer and output layer. Variables $b_{H}$ and $b_{O}$ represent the biases of the hidden layer and output layer.

CW-RNN divides the weight parameters $W_{H}$ and $W_{I}$ in Equation (1) into g rows, with each row corresponding to a module.

(3) $$W_{H}=\left(\begin{array}{l} W_{H_{1}}\\ \;\vdots \\ W_{H_{g}} \end{array}\right)W_{I}=\left(\begin{array}{l} W_{I_{1}}\\ \;\vdots \\ W_{I_{g}} \end{array}\right)$$

The differences between the hidden neurons of CW-RNN and standard RNN are as follows:

- The connections between modules in the hidden layer of CW-RNN only exist from modules with larger clock periods to those with smaller clock periods. Therefore, the hidden layer weight $W_{H}$ forms an upper triangular matrix, and $W_{H_{i}}$ can be represented as:
  (4) $$\left\{0_{1},\ldots ,0_{i-1},W_{H_{i,i}},\ldots ,W_{H_{i,g}}\right\}$$
- The activation of modules in the hidden layer of CW-RNN is controlled by clocks. At time t, only the modules for which t is divisible by $T_{i}\in \left\{T_{1},\ldots ,T_{g}\right\}$ will be activated, and the neurons within those modules will be activated. Therefore, $W_{H}$ can be expressed as a piecewise function:
  (5) $$W_{H_{i}}=\{\begin{array}{cc} W_{H_{i}} & if\left(t\;\mathrm{mod}\;T_{i}\right)=0\\ 0 & otherwise \end{array}$$

By substituting Equation (5) into Equation (1) for calculation, the modules activated at time t will update their output $y_{H}^{\left(t\right)}$, while the inactive modules will output their state values at time t−1, denoted as $y_{H}^{\left(t-1\right)}$.

The backpropagation calculation of CW-RNN still uses a time-based backpropagation algorithm. However, similar to the forward propagation, the backpropagation only affects the activated modules at time t. Only the parameters of the activated modules are updated, while the inactive modules continue to use the parameter values from time t−1. By adopting a modular structure and setting clock periods, each module in CW-RNN can process data at different times. By jointly outputting the long- and short-term modules, the output can learn both recent information from high-frequency update modules and long-term memory retained in low-frequency update modules, thereby enhancing its memory capabilities. Meanwhile, only a small number of neurons in CW-RNN participate in weight updates, which speeds up the training process. Therefore, the CW-RNN network structure is more suitable for network security situation prediction.

### 3.2. Grey Wolf Optimization Algorithm

The Grey Wolf Optimization Algorithm (GWO) [27], proposed by Mirjalili et al. in 2014, simulates the hunting process of wolf packs to gradually approach the target. The algorithm is divided into four stages: dividing the wolf pack into levels, tracking the prey, surrounding the prey, and attacking the prey.

Dividing the wolf pack into levels: The highest-ranking leader wolf, the second-ranking wolf, the third-ranking wolf, and the common wolf.

Tracking the prey: In nature, wolf packs track their prey before hunting. Then, they spread out according to the arrangement of the alpha wolf and prepare to attack the prey. This stage is called tracking the prey, and it is mapped to the GWO algorithm as the initialization of the wolf pack.

Surrounding the prey: After entering the hunting phase, the wolf pack will chase the prey and try to surround it. The alpha wolf leads the chase, and the other wolves follow around it. This process is mathematically modeled in the GWO algorithm as:

(7) $$\vec{D}=|\vec{C}\cdot {\vec{X}}_{p}\left(t\right)-\vec{X}\left(t\right)|$$

(7) $$\vec{X}\left(t+1\right)={\vec{X}}_{p}\left(t\right)-\vec{A}\cdot \vec{D}$$

At this stage, $\vec{D}$ represents the distance between the grey wolf and the target prey, ${\vec{X}}_{p}$ represents the position of the prey, $\vec{X}$ represents the current position of the wolf and is the current iteration number, $\vec{C}$ and $\vec{A}$ are coefficient vectors, which are defined as follows:

(8) $$\vec{A}=2\vec{a}\cdot \vec{r_{1}}-\vec{a}$$

(9) $$\vec{C}=2\cdot \vec{r_{2}}$$

The values in $\vec{r_{1}}$ and $\vec{r_{2}}$ are random numbers within the range of [0,1], with $\vec{a}$ linearly decreasing from 2 to 0.

Prey attack: During the attack on prey, the wolf pack surrounds the prey to force it to stop, and then the leader wolf commands the pack to attack. Wolf ω approaches wolf α, wolf β, and wolf δ using Equations (10)–(12), and obtains the three positions after moving, which are then averaged. The pack updates their positions based on wolf A, wolf α, and wolf A, and the mathematical model for this stage is:

(10) $${\vec{D}}_{\alpha }=|{\vec{C}}_{1}\cdot {\vec{X}}_{\alpha }-\vec{X}|,{\vec{D}}_{\beta }=|{\vec{C}}_{2}\cdot {\vec{X}}_{\beta }-\vec{X}|,{\vec{D}}_{\delta }=|{\vec{C}}_{3}\cdot {\vec{X}}_{\delta }-\vec{X}|$$

(11) $${\vec{X}}_{1}={\vec{X}}_{\alpha }-{\vec{A}}_{1}\cdot ({\vec{D}}_{\alpha }),{\vec{X}}_{2}={\vec{X}}_{\beta }-{\vec{A}}_{2}\cdot ({\vec{D}}_{\beta }),{\vec{X}}_{3}={\vec{X}}_{\delta }-{\vec{A}}_{3}\cdot (\vec{D_{\delta }})$$

(12) $$\vec{X}\left(t+1\right)=\frac{{\vec{X}}_{1}+{\vec{X}}_{2}+{\vec{X}}_{3}}{3}$$

Among them, ${\vec{X}}_{\alpha }$, ${\vec{X}}_{\beta }$, and ${\vec{X}}_{\delta }$, respectively, represent the positions of α wolf, β wolf, and δ wolf in the solution space.

- *Algorithm Selection and Rationale*

In addition to the Grey Wolf Optimization (GWO), we included Particle Swarm Optimization (PSO) [28] and the Genetic Algorithm (GA) [29] as comparison algorithms for optimizing the Clockwork Recurrent Neural Network (CW-RNN). PSO has garnered significant research attention and has demonstrated promising performance in neural network hyperparameter optimization, showcasing its potential for superior adaptability. Notably, PSO is well-suited for problems with real-valued encodings, which aligns with the continuous nature of neural network hyperparameter optimization [30].

As for the Genetic Algorithm (GA), it is a widely recognized optimization technique that employs principles inspired by biological evolution. GA operates on a population of potential solutions, utilizing genetic operators such as selection, crossover, and mutation to search for optimal solutions. Its ability to explore a diverse solution space and handle complex problems makes it a relevant choice for comparison in our study.

In contrast, we did not include Ant Colony Optimization (ACO) [31] in our comparison due to several factors. Firstly, ACO is more suited for problems with discrete encodings, whereas neural network hyperparameter optimization involves continuous variables. Secondly, our preliminary experiments revealed that ACO did not demonstrate a comparable performance to GWO in network security situational awareness tasks.

While ACO may possess advantages in specific optimization problems, our research focus centers around a meaningful comparison between GWO, PSO, and GA. These three algorithms, GWO, PSO, and GA, are all swarm intelligence-based optimization methods that have been extensively researched and applied in the field of network security [32]. Therefore, by evaluating their performance collectively, we can gain valuable insights into the effectiveness of swarm intelligence-based approaches in our research domain.

## 4. GWO-Based Situation Prediction Algorithm Using CW-RNN

In this section, we present our novel approach for optimizing the CW-RNN network using the Grey Wolf Optimization (GWO) algorithm. While the CW-RNN network structure and concepts have been previously introduced, the optimization method using GWO is our original contribution. We propose to optimize the hyperparameter combination, including the number of hidden layer neurons $h_{size}$, learning rate learning_rate, iteration times epochs, and batch size batch_size. These hyperparameters have a significant impact on the performance and convergence speed of the model.

Firstly, the number of neural units in the CW-RNN hidden layer refers to the quantity of neurons in that layer. A suitable number of hidden layer neural units can help the model capture the complex relationships and patterns within the data. By optimizing and finding the optimal number of neural units, the model’s expressive power and prediction accuracy can be enhanced.

Secondly, the learning rate represents the step size of parameter updates in the model. A smaller learning rate can lead to a more stable convergence but may result in a slower convergence speed. On the other hand, a larger learning rate may cause oscillation or failure to converge during the optimization process. By optimizing and determining the most appropriate learning rate, a balance can be achieved between model stability and training speed.

The Iteration times refer to the total number of parameter updates performed by the model during the training process. An appropriate number of iterations allows the model to learn the features and patterns within the data more comprehensively. Too few iterations may prevent the model from reaching its optimal performance, while excessive iterations may lead to overfitting. Therefore, through optimization, the appropriate number of iterations can be determined to improve the model’s generalization ability and prediction performance.

Lastly, the batch size represents the number of samples inputted into the model during each iteration. A larger batch size can improve the training efficiency of the model but may cause it to get stuck in local optima or overfit. Conversely, a smaller batch size allows the model to better capture fine-grained features among the samples but may result in a slower training process. By optimizing and determining the optimal batch size, a balance can be achieved between training efficiency and model performance.

Different combinations of hyperparameters have different effects on the final prediction accuracy. In order to avoid the arbitrariness of hyperparameter settings, GWO is used to optimize the hyperparameter combination ($h_{size}$, learning_rate, epochs, batch_size) of CW-RNN. In intelligent swarm optimization algorithms similar to GWO, fitness is an important indicator for measuring individual performance. It can select individuals based on different degrees of adaptability, and individuals with higher fitness values are more likely to become leaders and correspond to better solutions. The fitness function, also known as the evaluation function, measures the degree of excellence of individuals in the group. In the situation prediction model, the fitness function is set as follows:

(13) $$fitness=\frac{1}{G}\sum_{g-1}^{G}{\left(y_{g}^{'}-y_{g}\right)}^{2}$$

where $y_{g}^{'}$ represents the model output value, $y_{g}$ represents the true value of the data, and G represents the total amount of data.

The following are pseudocodes for our proposed gray wolf algorithm and CW-RNN network design (Algorithms 1 and 2):
**Algorithm 1:** Pseudocode of Grey Wolf Algorithm# Grey Wolf algorithm parameter initialization 1. population_size = 20 2. max_iterations = 15 3. lower_bound = [8, 10^−4^, 100, 16] 4. upper_bound = [200, 5 × 10^−^, 600, 128] # Grey Wolf algorithm population initialization 5. population = initialize_population(population_size, lower_bound, upper_bound) # Iteratively search for the optimal solution 6. for iteration in range(max_iterations): 7.        fitness = evaluate_fitness(population) # Calculate the fitness value 8.        update_positions(population, fitness) # Update wolf pack position 9. best_solution = get_best_solution(population) # Get the optimal solution # Output the hyperparameters of the optimal solution 10. best_hyperparameters = decode_parameters(best_solution)

**Algorithm 2:** CW-RNN Network Pseudocode# CW-RNN network parameter initialization num_hidden_units = …learning_rate = …num_iterations = …batch_size = …
# CW-RNN network model construction
5.model = build_cw_rnn_model(num_hidden_units, learning_rate)
# CW-RNN network training
6.for iteration in range(num_iterations):
# Get the current training batch data
7.batch_data = get_next_batch(batch_size)
# forward pass
8.predictions = model.forward_pass(batch_data)
# Compute the loss function
9.loss = calculate_loss(predictions, batch_data)
# Backpropagation
10.gradients = model.backward_pass(loss)
# Update network parameters
11.model.update_parameters(gradients)
# CW-RNN network prediction
12.test_data = load_test_data()13.predictions = model.predict(test_data)


**Figure 2:**
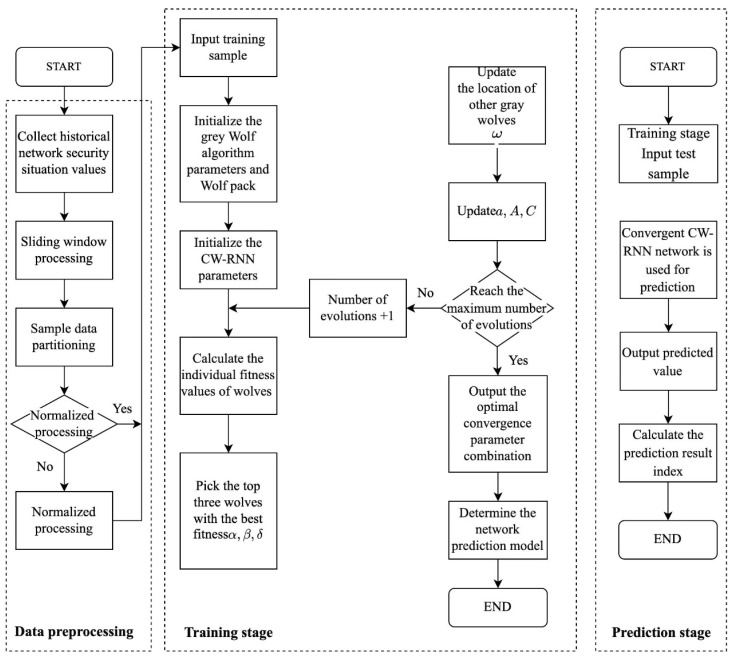
Flowchart of network security posture prediction algorithm based on GWO optimized CW-RNN.

To illustrate the process, we provide a flowchart in Figure 2, depicting the network security situation prediction algorithm based on the GWO-optimized CW-RNN. The algorithm consists of two phases: the training phase and the prediction phase.

In order to optimize the CW-RNN network using the Grey Wolf Optimization (GWO) algorithm, we follow a series of design steps. The training phase consists of the following specific steps:

Step 1: Collect historical network security situation values.
Step 2: Process the collected network security situation values using sliding window technology.
Step 3: Divide the data set into training set and test set.
Step 4: Check whether the data has been normalized. If it has been normalized, proceed to Step 6; otherwise, proceed to Step 5.
Step 5: Normalize the historical network security situation values.
Step 6: Input the training set.
Step 7: Initialize the Grey Wolf algorithm parameters and wolf pack. The parameters of the Grey Wolf algorithm include the total number of wolves, the maximum number of evolution iterations, etc. Convert the number of hidden layer neurons $h_{size}$, learning_rate, iteration times epochs, and batch_size for the clock-cycle Recurrent Neural Network into the parameter coordinates of the wolf pack individuals’ positions, and set upper and lower limits.
Step 8: Initialize the parameters of the clock-cycle Recurrent Neural Network. The parameters of the neural network include the number of input neurons, the number of output neurons, and the clock-cycle of the network.
Step 9: SCalculate the fitness value of each individual in the wolf pack. The fitness function is the mean square error between the predicted value and the true value.
Step 10: Compare the fitness values of each grey wolf and select the top three wolves with the best fitness values as α, β, and δ.
Step 11: Update the positions of other grey wolves D based on α, β, and δ.
Step 12: Update the convergence factor α, convergence coefficient vector $\vec{A}$, and coordination coefficient vector $\vec{C}$ in the Grey Wolf algorithm.
Step 13: Check whether the maximum number of evolution iterations has been reached. If it has, proceed to Step 14; otherwise, increase the iteration number by one and proceed to Step 9.
Step 14: Output the position coordinates of α, which is the optimal parameter combination ($h_{size}$, learning_rate, epochs, batch_size) of the clock-cycle Recurrent Neural Network.
Step 15: Bring the optimal parameter combination ($h_{size}$, learning_rate, epochs, batch_size) back to the clock-cycle Recurrent Neural Network and train it to obtain an optimized network security situation prediction model.

The specific steps of the prediction phase are as follows:

Step 1: Input the test sample.
Step 2: Predict using the optimized converged network security situation prediction model.
Step 3: Output the prediction value.
Step 4: Calculate the prediction result indicators, including the mean relative error, mean absolute error, and minimum mean square error.

## 5. Experimental Simulation and Analysis

### 5.1. Datasets and Experiments

In order to evaluate and validate the performance of the Clockwork Recurrent Neural Network (CW-RNN) in network security situation prediction, this study selected three commonly used network security datasets for experimentation, namely CICIDS2017, UNSW-NB15, and NSL-KDD. These datasets are widely applied and extensively validated and researched in the field of network intrusion detection. Specifically, these datasets provide a large number of network traffic records, including various types of normal traffic and different types of network intrusion behaviors, ensuring the comprehensiveness and reliability of the experiments to evaluate the performance and generalization ability of the proposed CW-RNN model on different datasets. Using these datasets, this paper aims to provide a comprehensive assessment of the application and performance of the CW-RNN model in real network security environments.

The experiments in this study were conducted using a server provided by the university. The server was equipped with 48 NVIDIA GeForce RTX 2080 Ti graphics cards. The software used for the experiments was TensorFlow 2.5.0.

### 5.2. CICIDS2017 Dataset

The experimental data used in this study is from the CICIDS2017 dataset, which is a comprehensive and widely used dataset in network intrusion detection research. Collected by the Canadian Institute for Cybersecurity (CIC), the CICIDS2017 dataset provides an extensive collection of network traffic records that reflect real-world scenarios.

The CICIDS2017 dataset encompasses a significant volume of network traffic data, with a size of approximately 8.3G. It comprises various network traffic features, including packet-level information, flow-level information, and application-level information. The dataset captures a diverse range of attack scenarios, such as Denial-of-Service (DoS), Distributed Denial-of-Service (DDoS), probing, infiltration, and botnet activities. These attack scenarios are based on different network protocols and exhibit distinct attack strategies and behaviors. Furthermore, the dataset incorporates a substantial amount of normal traffic data, which represents typical network activities. Each network traffic record in the CICIDS2017 dataset is labeled with its corresponding attack type or labeled as normal traffic. These labels provide ground truth information for evaluating the performance of intrusion detection models.

By utilizing the CICIDS2017 dataset, we aim to demonstrate the effectiveness of our proposed optimization approach for the clock-cycle Recurrent Neural Network in network security situation prediction tasks. The dataset’s large size and diverse network traffic characteristics ensure the generalizability and robustness of the proposed optimization model, enabling us to evaluate its performance comprehensively. Overall, the CICIDS2017 dataset provides a realistic and challenging environment for evaluating network security prediction models, serving as a valuable resource for researchers and practitioners in the field of network intrusion detection and network security.

- *Data Preprocessing*

Based on the situational evaluation, the data for network security situational prediction experiments were obtained using the situational evaluation method described in Chapter 3 to evaluate the situational values of the Friday dataset in the CICIDS-2017 traffic dataset. The situational values obtained from the situational evaluation were used to form historical and current situational values in chronological order. The data samples were divided according to Table 2, resulting in a total of 5019 samples, which were divided into training and testing sets in a 7:3 ratio.

- *Simulation Analysis of Optimal Parameter Combination for the Model*

In the CW-RNN network, the selection of model parameters is mostly based on experience and manual settings, which has a certain degree of randomness and affects the performance of the model. To obtain a good model, multiple experiments are required to determine the appropriate parameters. In this paper, the GWO algorithm was used to optimize the CW-RNN parameters. The specific optimization parameters include the number of neural units in the CW-RNN hidden layer, learning rate, iteration times, and batch size. Other key parameter settings are as follows: 10 input neurons, 1 output neuron, initialization of 20 populations in GWO, a maximum of 15 iterations, and the lower limit of the optimization parameters is [8, 10^−4^, 100, 16], and the upper limit is [200, 5 × 10^−2^, 600, 128]. The fitness function is mean squared error.

**Figure 3:**
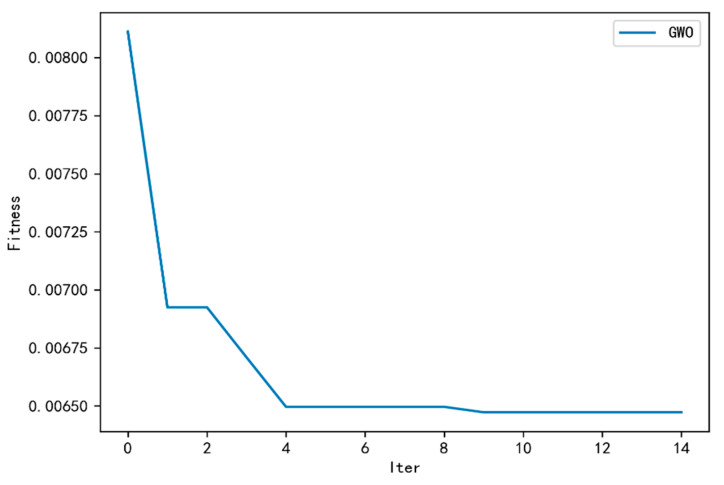
Convergence graph of GWO optimized CW-RNN.

**Figure 4:**
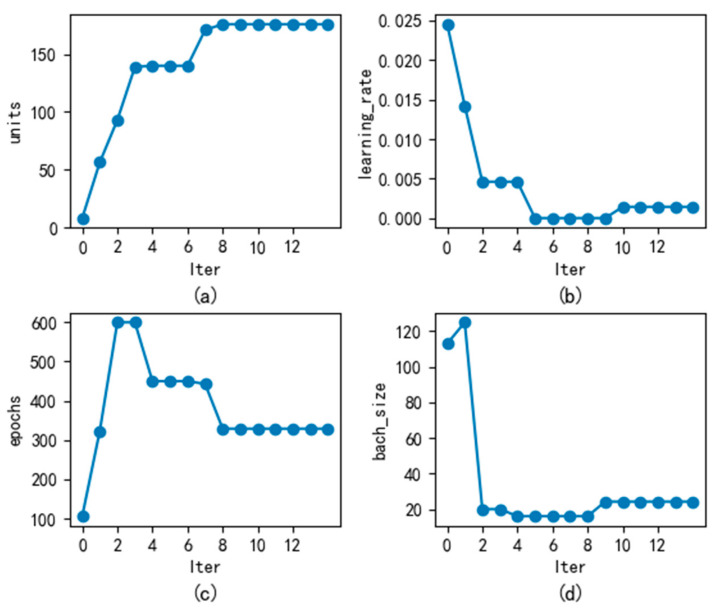
GWO optimization of CW-RNN for parameter variation.

Figure 3 shows the convergence graph of the fitness function value, which is the error function optimized by GWO-CW-RNN. From the figure, it can be seen that the error function value drops rapidly in the first iteration and continues to decrease. After the ninth iteration, it gradually converges. At this time, Figure 4 shows the parameter change graph of GWO-CW-RNN optimization, where Figure 4a shows the convergence graph of the number of neural units in the hidden layer with the algorithm iteration. Figure 4b shows the convergence graph of the learning rate with the algorithm iteration. Figure 4c shows the convergence graph of the CW-RNN training iteration times with the algorithm iteration. Figure 4d shows the convergence graph of the network input with the algorithm iteration. From the figure, it can be seen that the number of neural units in the hidden layer finally converges to 176, the learning rate is 0.001437, the CW-RNN training iteration times are 329, and the network input is 24.

Therefore, the best hyperparameters were obtained to correct the structure of the proposed situational prediction model in this chapter, and the optimal parameter combination of the model was obtained.

- *Analysis of Network Situation Prediction Results*

**Figure 5:**
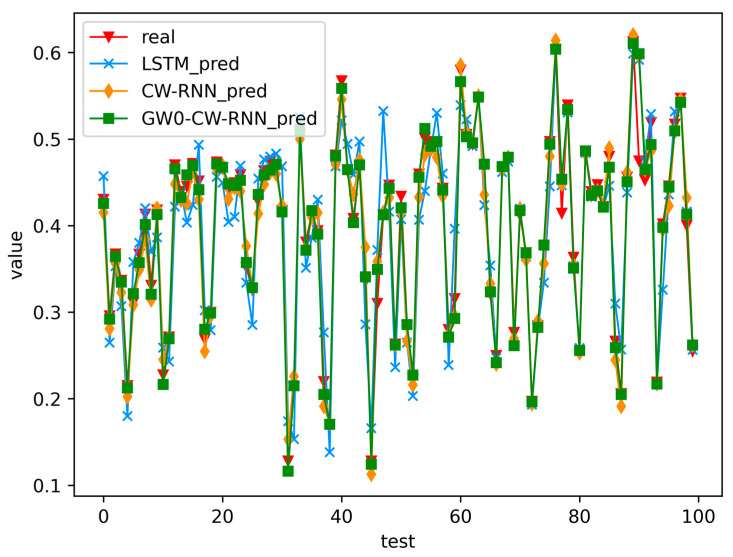
Comparison of the prediction results of the three methods on the test set.

Here, we present the experimental results and analysis of the proposed GWO-optimized CW-RNN, compared with the original CW-RNN and LSTM methods, on the test dataset. The prediction results of the three methods are illustrated in Figure 5. To provide a more intuitive and effective evaluation of the proposed network security situation prediction method in this chapter, we calculate the Mean Absolute Percentage Error (MAPE), Mean Absolute Error (MAE), and Minimum Mean Square Error (MSE) of the predictions from the three methods on the test dataset. The detailed calculation process is shown in Equations (14)–(16), where $y_{t}$ represents the ground truth values and $\hat{y_{t}}$ represents the predicted values. The comparative results are summarized in Table 3.

(14) $$MAPE=\frac{1}{n}\sum_{t=1}^{n}\left|\frac{y_{t}-\hat{y_{t}}}{y_{t}}\right|\times 100\%$$

(15) $$MAE=\frac{1}{n}\sum_{t=1}^{n}\left|y_{t}-\hat{y_{t}}\right|$$

(16) $$MSE=\frac{1}{n}{\sum_{t=1}^{n}\left(y_{t}-\hat{y_{t}}\right)}^{2}$$

From Figure 5, it can be observed that LSTM generally captures the overall trend of the data, while compared to LSTM, the prediction performance of the CW-RNN network is more pronounced. This is because CW-RNN exhibits a stronger advantage in learning short-term dependent data features compared to LSTM. Furthermore, the GWO-optimized CW-RNN, benefiting from the parameter optimization performed by GWO, yields slightly better prediction results compared to the original CW-RNN network.

Firstly, by examining the Mean Absolute Percentage Error (MAPE) metric, we can see that the GWO-CW-RNN method outperforms the others in network security situation prediction. Its MAPE value of 4.6153 is significantly lower than the values of 5.3840 for LSTM and 4.8902 for CW-RNN. This indicates that the GWO-CW-RNN method is capable of predicting network security situations more accurately with smaller relative errors.

Furthermore, the Mean Absolute Error (MAE) and Mean Squared Error (MSE) metrics also support this conclusion. The GWO-CW-RNN method achieves an MAE of 0.03473, which is noticeably lower than the values of 0.03952 for LSTM and 0.03825 for CW-RNN. Similarly, GWO-CW-RNN has an MSE of 0.00615, smaller than LSTM’s 0.00741 and CW-RNN’s 0.00692. This suggests that the GWO-CW-RNN method exhibits smaller average errors and better fitting performance in predicting network security situations.

In summary, the experimental results demonstrate that the GWO-CW-RNN method outperforms LSTM and CW-RNN in network security situation prediction. By optimizing the parameters of the Clockwork Recurrent Neural Network using the Grey Wolf Optimization algorithm, this method achieves more accurate predictions. These results serve as a foundation for further converting the model evaluation metrics to accuracy, precision, recall, and F1 score in a classification model, enabling a more comprehensive assessment of the performance of network security situation prediction methods.

- *Comparative Experimental Results Analysis of Optimization Algorithms*

In this study, we have transformed the evaluation metrics of the regression model into a classification model for comparison, aiming to better reflect the performance and effectiveness of the optimized model in practical applications. This transformation allows us to address the network security situation prediction problem as a decision problem with practical significance, rather than a purely numerical fitting problem. By categorizing the predicted results into different security levels and taking corresponding measures, we can more accurately address real-world situations.

To achieve this transformation, we first set thresholds to categorize the network security situation values into different classes. Subsequently, we convert the real values and predicted values into corresponding class labels. Then, we calculate evaluation metrics such as accuracy, precision, recall, and F1-score based on the predicted labels and real labels. The results of these evaluation metrics are reported and compared with other models.

Through this transformation, we can comprehensively assess the performance of the optimized model in classification problems, enabling a better judgment of its advantages in practical applications. Moreover, this approach allows us to compare the performance of different models using the same metrics, thereby determining whether the optimized model demonstrates a significant improvement. The transformation to a classification model provides us with a more comprehensive evaluation perspective and a better understanding of the practical decision-making capabilities of the optimized model.

To provide a more intuitive and effective evaluation of the proposed network security situation prediction method in this chapter, we assess the performance of the model using the following four metrics: accuracy, precision, recall, and F1-score. The detailed calculation process is shown in Equations (17)–(20).

TP (True Positive): The number of instances correctly predicted as positive. It refers to the cases where the true value and the predicted value are both positive.

TN (True Negative): The number of instances correctly predicted as negative. It refers to the cases where the true value and the predicted value are both negative.

FP (False Positive): The number of instances incorrectly predicted as positive. It refers to the cases where the true value is negative, but the predicted value is positive.

FN (False Negative): The number of instances incorrectly predicted as negative. It refers to the cases where the true value is positive, but the predicted value is negative.

These parameters help us understand the performance of the model in terms of correctly identifying positive and negative instances, as well as the occurrences of false positives and false negatives.

(17) $$Accuracy=\frac{TP+TN}{TP+FP+TN+FN}$$

(18) $$Precision=\frac{TP}{TP+FP}$$

(19) $$Recall=\frac{TP}{TP+FN}$$

(20) $$F_{1}=2\times \frac{Precision\times Recall}{Precision+Recall}$$

In the comparative experiments of optimization algorithms on the CICIDS2017 dataset, we employed Grey Wolf Optimization (GWO), Particle Swarm Optimization (PSO), and Genetic Algorithm (GA) to optimize the Clockwork Recurrent Neural Network (CW-RNN). The following table presents the performance of each algorithm in terms of Accuracy, Precision, Recall, and F1-score:

The results shown in Table 4 indicate that the GWO algorithm outperforms other optimization algorithms in terms of accuracy, precision, recall, and F1-score. It achieved the highest performance with an accuracy of 0.95, precision of 0.91875, recall of 0.93, and F1-score of 0.92435. In comparison, PSO and GA also yielded relatively good results but were inferior to GWO.

Additionally, we tested the performance of the unoptimized CW-RNN model, which exhibited relatively lower values in all metrics. It achieved an accuracy of 0.88, precision of 0.84762, recall of 0.86, and F1-score of 0.85375. This further validates the effectiveness of optimization algorithms in enhancing the performance of the CW-RNN model.

In summary, through the comparative experiments of optimization algorithms, we observed that the GWO algorithm demonstrates excellent performance in optimizing the CW-RNN model, significantly improving its performance metrics, and providing an effective optimization solution for network security situational prediction. Next, we will further analyze the results of the model comparison experiments.

- *Analysis of Model Comparison Experiment Results*

**Figure 6:**
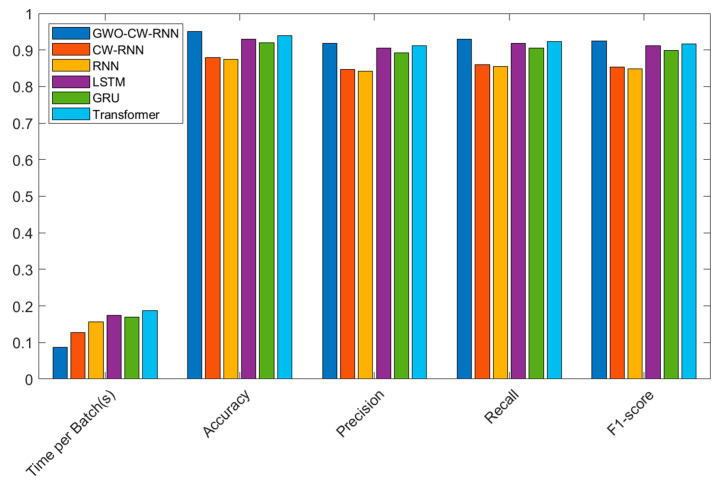
Experimental results of model performance comparison on the CICIDS2017 dataset.

In the comparative experiments of models on the CICIDS-2017 dataset, we tested the Grey Wolf Optimization (GWO) optimized Clockwork Recurrent Neural Network (CW-RNN), CW-RNN without optimization, Recurrent Neural Network (RNN), Long Short-Term Memory (LSTM), Gated Recurrent Unit (GRU), and Transformer model. The following table presents the performance of each model in terms of time per batch (s), accuracy, precision, recall, and F1-score:

From the experimental results shown in Table 5 and Figure 6, the following observations can be made:

The GWO-optimized CW-RNN exhibits the best performance. It has a significantly faster running time (0.087 s) compared to other models, along with high accuracy (0.95000), precision (0.91875), recall (0.93000), and F1-score (0.92435). This demonstrates the remarkable improvement achieved by the GWO-optimized CW-RNN in network security situational prediction.

The unoptimized CW-RNN model has a slightly slower running time compared to the GWO-optimized model (0.127 s) but still demonstrates high performance. It has high accuracy (0.88000), precision (0.84762), recall (0.86000), and F1-score (0.85375). However, its performance is slightly lower compared to the GWO-optimized model.

The Recurrent Neural Network (RNN) has a relatively slower running time (0.156 s) and lower accuracy (0.87500), precision (0.84211), recall (0.85500), and F1-score (0.84850).

Both the Long Short-Term Memory (LSTM) and Gated Recurrent Unit (GRU) have slightly slower running times (0.174 s and 0.169 s, respectively), but they exhibit a high accuracy, precision, recall, and F1-score.

The Transformer model has the slowest running time (0.188 s) but still maintains high accuracy, precision, recall, and F1-score.

In conclusion, the GWO-optimized CW-RNN demonstrates the best performance and faster running speed on the CICIDS-2017 dataset, showcasing its advantages in network security situational prediction. This confirms the effectiveness of our proposed model and supports the application value of GWO optimization in Clockwork Recurrent Neural Networks. Furthermore, our CW-RNN model holds potential for real-time monitoring of large-scale network traffic in sensor networks due to its fast execution speed, which contributes to reducing runtime.

- *Comparison of the Time Complexity of the Models*

In this section, we present a comparison of the time complexity of various models, which is an essential aspect to consider when selecting the most efficient model for practical applications. We have evaluated and calculated the time complexities of different models, including our proposed optimized CW-RNN model and several comparison models. The time complexities are presented in terms of big O notation, with simplified expressions by ignoring lower-order terms and constants. This allows us to focus on the dominant factors that impact the computational efficiency of the models. The table below provides an overview of the time complexities of each model.

From Table 6, we can draw the following conclusions. The time complexity of our proposed model has the same order of magnitude as that of the RNN model, but our algorithm has an additional parameter M, which can be increased by increasing the number of hidden layer modules. Reduce time complexity. This means that our algorithm can be parallelized to improve efficiency, while the RNN model is limited to serialized calculations. Furthermore, both the LSTM model and the GRU model involve a large number of matrix multiplications and additions, as well as activation functions and gating mechanisms, which make their time complexity an order of magnitude higher than our algorithm. Although the Transformer model can parallelize the calculation, it needs to calculate the scaled dot product attention, which makes its time complexity grow quadratically with the length of the input sequence, which is unacceptable for long sequences. To sum up, we can conclude that our algorithm outperforms the comparison algorithm in terms of time complexity, especially for long sequences, our algorithm has higher efficiency.

### 5.3. UNSW-NB15 Dataset

The UNSW-NB15 dataset is a comprehensive network traffic dataset collected by the University of New South Wales (UNSW), specifically designed for network intrusion detection research. This dataset comprises a large-scale collection of network traffic records, consisting of approximately 2.5 million instances. The dataset provides an extensive range of network traffic features, including packet-level information, flow-level information, and application-level information. It offers a comprehensive view of network communication and facilitates in-depth analysis of network behaviors.

The UNSW-NB15 dataset encompasses various attack scenarios, such as Denial-of-Service (DoS), Distributed Denial-of-Service (DDoS), probing, infiltration, and botnet activities. Each attack scenario exhibits distinct characteristics, reflecting different attack strategies and behaviors. In addition to the attack scenarios, the dataset also contains normal traffic data, representing typical network activities. This diversity enables researchers to evaluate intrusion detection models in a realistic and challenging environment. To facilitate model evaluation, the records in the UNSW-NB15 dataset are labeled, providing ground truth information for intrusion detection. These labels allow researchers to assess the accuracy and performance of their models in detecting and classifying network attacks.

The UNSW-NB15 dataset, with its large scale and diverse network traffic records, serves as a valuable resource for developing and evaluating network security prediction models. It offers researchers the opportunity to explore innovative techniques and improve the detection and prediction capabilities of network security systems.

- *Comparative Experimental Results Analysis of Optimization Algorithms*

**Figure 7:**
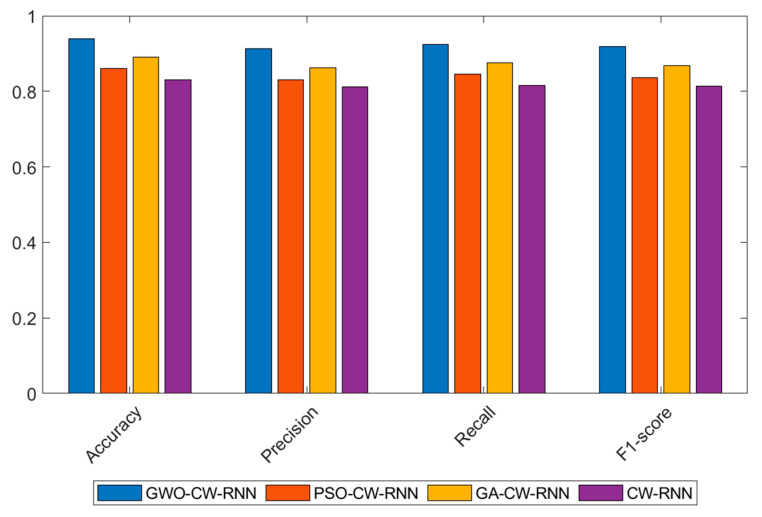
Experimental results of performance comparison of optimization algorithms on the UNSW-NB15 dataset.

In the comparative experiments of optimization algorithms on the UNSW-NB15 dataset, we tested the Grey Wolf Optimization (GWO), Particle Swarm Optimization (PSO), and Genetic Algorithm (GA) for optimizing the Clockwork Recurrent Neural Network (CW-RNN). The following table presents the performance of each model in terms of accuracy, precision, recall, and F1-score:

The following conclusions can be drawn from the experimental results shown in Table 7 and Figure 7:

The GWO-optimized network achieves the best performance in terms of accuracy, precision, recall, and F1-score. It has an accuracy of 0.94, precision of 0.91234, recall of 0.925, and F1-score of 0.91861. This indicates that the Grey Wolf Optimization algorithm performs well in optimizing the Clockwork Recurrent Neural Network, significantly improving the predictive performance of the network.

The CW-RNN without any optimization shows relatively lower performance in all metrics, with an accuracy of 0.83, precision of 0.81225, recall of 0.815, and F1-score of 0.81362. Compared to the optimized models, its performance is lower, further confirming the importance of optimization algorithms in model performance.

The PSO and GA-optimized networks exhibit moderate performance in all metrics, with accuracies of 0.86 and 0.89, respectively. These optimization algorithms achieve some performance improvements compared to the unoptimized model, but there is still a gap compared to the Grey Wolf Optimization algorithm.

In summary, in the comparative experiments of optimization algorithms on the UNSW-NB15 dataset, the GWO-optimized Clockwork Recurrent Neural Network demonstrates the best performance with high accuracy, precision, recall, and F1-score. This indicates that the Grey Wolf Optimization algorithm has a good effect on optimizing the network for network security situational prediction tasks, enhancing the model’s generalization ability and robustness.

- *Analysis of Model Comparison Experiment Results*

**Figure 8:**
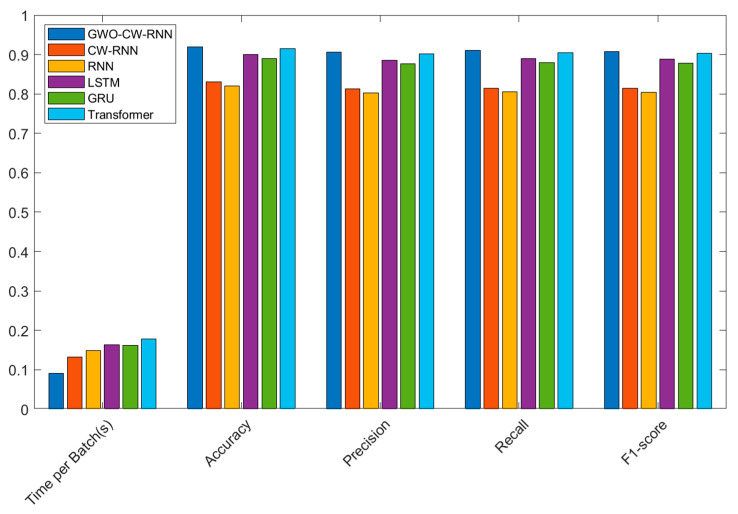
Experimental results of model performance comparison on the UNSW-NB15 dataset.

In the comparative experiments on the UNSW-NB15 dataset, we evaluated the performance of different models for network security situational prediction. The following table presents the performance of each model in terms of time per batch (s), accuracy, precision, recall, and F1-score:

From the experimental results shown in Table 8 and Figure 8, the following conclusions can be drawn:

The GWO-CW-RNN model is the Clockwork Recurrent Neural Network model optimized using the Grey Wolf Optimization algorithm. This model exhibits the best performance and has a relatively short running time on the UNSW-NB15 dataset. Specifically, the GWO-CW-RNN model achieves an accuracy of 0.92000, a precision of 0.90575, a recall of 0.910, and an F1-score of 0.90786. Furthermore, this model has a time per batch of 0.091 s, making it faster compared to other models.

In contrast, the unoptimized CW-RNN model shows relatively lower performance in terms of accuracy, precision, recall, and F1-score. It has a time per batch of 0.132 s, slightly longer than the GWO-CW-RNN model. Additionally, the traditional RNN, LSTM, GRU, and Transformer models perform between the GWO-CW-RNN model and CW-RNN model in terms of accuracy and other metrics, and they have relatively longer time per batch.

In summary, the GWO-CW-RNN model, which is the Clockwork Recurrent Neural Network model optimized using the Grey Wolf Optimization algorithm, achieves higher accuracy and performance on the UNSW-NB15 dataset, while having a shorter running time. This indicates that the model has a significant advantage in network security situational prediction tasks.

### 5.4. NSL-KDD Dataset

The NSL-KDD dataset is a widely used network intrusion detection dataset, derived from the original KDD Cup 1999 dataset. This dataset has undergone extensive preprocessing to address various limitations and enhance its usefulness for intrusion detection research.

The NSL-KDD dataset encompasses a diverse set of network traffic features, providing a comprehensive representation of network communication. It includes information at the packet level, connection level, and application level, enabling researchers to analyze network behaviors from multiple perspectives. In terms of attack types, the NSL-KDD dataset covers a range of scenarios, including Denial-of-Service (DoS), probing, Remote-to-Local (R2L), and User-to-Root (U2R) attacks. Each attack type exhibits unique characteristics, reflecting different intrusion strategies and techniques. By incorporating multiple attack types, the dataset offers a realistic and challenging environment for evaluating intrusion detection models.

Similar to the UNSW-NB15 dataset, the NSL-KDD dataset provides labeled records, allowing researchers to evaluate the performance of intrusion detection models accurately. These labels provide ground truth information for classifying network traffic instances as either normal or malicious, facilitating the assessment of model accuracy and effectiveness. With its comprehensive set of network traffic features, diverse attack types, and labeled records, the NSL-KDD dataset serves as an important benchmark for testing the performance of network security prediction models. Researchers can leverage this dataset to develop and evaluate innovative intrusion detection techniques, improving the detection and prediction capabilities of network security systems.

In summary, the NSL-KDD dataset, derived from the original KDD Cup 1999 dataset, offers a collection of network traffic records with diverse features and attack types. Its labeled records provide valuable ground truth information for evaluating intrusion detection models and serve as a benchmark for assessing the performance of network security prediction models.

- *Comparative Experimental Results Analysis of Optimization Algorithms*

**Figure 9:**
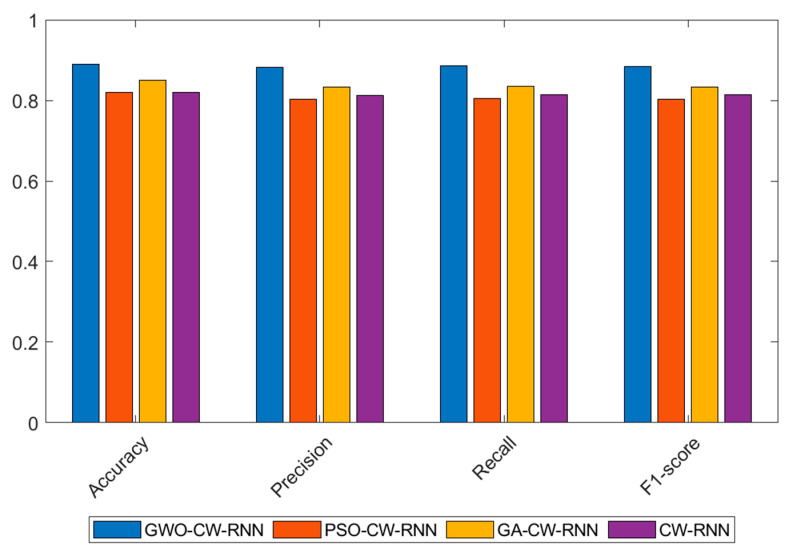
Experimental results of performance comparison of optimization algorithms on the NSL-KDD dataset.

In the comparative experiments of optimization algorithms on the NSL-KDD dataset, the results are as follows:

Based on the analysis of the experimental results shown in Table 9 and Figure 9:

The GWO-CW-RNN model exhibits the best performance, with an accuracy of 0.89000, a precision of 0.88234, a recall of 0.885, and an F1-score of 0.88361. This indicates that the Grey Wolf Optimization algorithm performs well in optimizing Recurrent Neural Networks for the NSL-KDD dataset.

The models optimized using the Particle Swarm Algorithm and the Genetic Algorithm also perform well in terms of accuracy, precision, recall, and F1-score. However, their performance is slightly inferior to the GWO-CW-RNN model.

The unoptimized Clockwork Recurrent Neural Network shows mediocre performance on the NSL-KDD dataset, with an accuracy of 0.82000, a precision of 0.81225, a recall of 0.815, and an F1-score of 0.81362. Its performance is lower compared to the optimized models.

In conclusion, the experimental results further demonstrate the effectiveness and superiority of the Grey Wolf Optimization algorithm in optimizing Clockwork Recurrent Neural Networks, particularly achieving good performance on the NSL-KDD dataset.

- *Analysis of Model Comparison Experimental Results*

**Figure 10:**
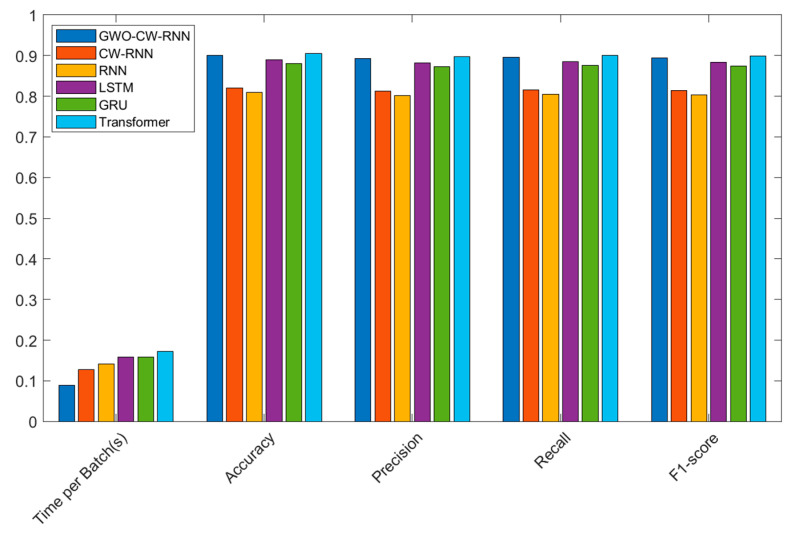
Experimental results of model performance comparison on the NSL-KDD dataset.

In the comparative experiments of models on the NSL-KDD dataset, the results are as follows:

Based on the analysis of the experimental results shown in Table 10 and Figure 10:

The GWO-CW-RNN model, which is a Clockwork Recurrent Neural Network model optimized using the Grey Wolf Optimization algorithm, demonstrates the best performance and shorter running time on the NSL-KDD dataset. The model achieves an accuracy of 0.90000, a precision of 0.89275, a recall of 0.895, and an F1-score of 0.89386. Additionally, it has a faster running time of 0.089 s per batch compared to other models.

In contrast, the unoptimized CW-RNN model shows relatively lower accuracy, precision, recall, and F1-score. Its running time per batch is 0.128 s. Traditional models such as RNN, LSTM, GRU, and Transformer perform between the GWO-CW-RNN model and the CW-RNN model in terms of performance metrics, and they have relatively longer running times per batch.

In conclusion, the GWO-CW-RNN model, optimized using the Grey Wolf Optimization algorithm, exhibits higher accuracy and performance on the NSL-KDD dataset, along with a shorter running time. This further validates the advantages of this model in the task of network security situation prediction.

## 6. Conclusions

In this study, we proposed a network security situation prediction method based on optimized Clockwork Recurrent Neural Networks to address the issue of low accuracy in existing methods when dealing with the temporal and nonlinear characteristics of network security situations. We designed Grey Wolf Optimization (GWO), Particle Swarm Optimization (PSO), and Genetic Algorithm (GA) to optimize the Clockwork Recurrent Neural Network, and through experiments, we selected GWO as the best optimization algorithm. Furthermore, we compared multiple models and demonstrated the advantages of the optimized Clockwork Recurrent Neural Network in terms of generalization capability. To validate the real-time performance of the model, we conducted experiments on multiple datasets and found that our network prediction model had the shortest execution time among all models, demonstrating its superior real-time performance. Therefore, our method is particularly suitable for monitoring large-scale network traffic in sensor networks.

However, our experiments were conducted on specific datasets, namely CICIDS2017, UNSW-NB15, and NSL-KDD datasets. Although these datasets are widely used in network security research, further research and validation are needed to assess the generalization capability of our method on other network environments and datasets. Different network environments may exhibit distinct characteristics and data distributions, thus necessitating the expansion of our experimental scope to verify the applicability of our method.

In practical applications, our optimized Clockwork RNN holds great potential for real-time monitoring and decision-making in network security. By accurately predicting the network security situation, appropriate measures can be taken to mitigate potential threats and ensure the security of network systems. For example, our model can be integrated into intrusion detection systems or security operation centers to provide timely alerts and facilitate proactive response strategies.

To further enhance the practicality and applicability of our approach, future research can focus on integrating additional features, such as network traffic analysis or contextual information, into the prediction model. Moreover, efforts can be made to develop user-friendly interfaces and tools that allow security professionals to easily deploy and utilize our optimized model in real-world scenarios. In summary, this study successfully addressed the issues of temporal and nonlinear characteristics in network security situation prediction through the use of GWO-optimized Clockwork Recurrent Neural Networks, and its superiority was validated on multiple datasets. Our method not only improves the accuracy of network security situation prediction but also possesses real-time performance and is suitable for sensor network monitoring. Future research can further explore other optimization algorithms and model structures to further enhance the performance of network security situation prediction.

## Figures and Tables

**Table 1:** Advantages compared to state-of-the-art research models.

Method	Advantages
Traditional methods	Our model outperforms Bayesian models and support vector machines in extracting temporal features of network security situations, demonstrating superior computational efficiency and real-time performance. It accurately predicts the evolving trends and changes in network security.
Neural network-based methods	In contrast to traditional back propagation neural networks, our model incorporates an internal memory mechanism, enabling better handling of time series data and avoiding performance degradation in network intrusion detection tasks. Compared to single Recurrent Neural Network variants such as LSTM or GRU, our model leverages the advantages of clock-cycle recurrence and periodic update mechanisms, comprehensively capturing complex and dynamic temporal features of network security situations, thereby enhancing prediction accuracy and performance.
Attention-based methods	Compared to methods utilizing the Dual Attention Mechanism and HHO-ResNeXt, our model offers greater flexibility and adaptability, effectively coping with dynamic and complex network security environments. By adjusting the length of the clock-cycle and the update mechanism, our model can adapt to different network security data and provide superior prediction accuracy. In comparison to Attention-CNN-BiGRU, our model places greater emphasis on extracting and modeling temporal features, thereby accurately predicting the development trends and changes in network security.
Transformer-based methods	Our model is better suited for handling time series data and exhibits improved real-time performance compared to Transformer models. Transformers often require significant computational resources and time for training and inference, limiting their application in real-time tasks. In contrast, our CC-RNN model, designed with clock-cycle recurrence, offers higher real-time performance.

**Table 2:** Sample data division.

Input Situation Information	Output Situation Information
*S*_1_, *S*_2_, …, *S*_10_	*S*_11_
*S*_11_, *S*_12_, …, *S*_20_	*S*_21_
…	…
*S_i_*, *S*_*i*+1_, …, *S*_*i*+9_	*S*_*i*+10_

**Table 3:** Prediction results on test set.

Method	MAPE	MAE	MSE
GWO-CW-RNN	4.6153	0.03473	0.00615
LSTM	5.3840	0.03952	0.00741
CW-RNN	4.8902	0.03825	0.00692

**Table 4:** Experimental results of performance comparison of optimization algorithms on the CICIDS2017 dataset.

Algorithm	Accuracy	Precision	Recall	F1-Score
GWO-CW-RNN	0.95	0.91875	0.93	0.92435
PSO- CW-RNN	0.93	0.87826	0.91	0.89378
GA- CW-RNN	0.94	0.88723	0.92	0.90328
CW-RNN	0.88	0.84762	0.86	0.85375

**Table 5:** Experimental results of model performance comparison on the CICIDS2017 dataset.

Model	Time per Batch (s)	Accuracy	Precision	Recall	F1-Score
GWO-CW-RNN	0.087	0.95000	0.91875	0.93000	0.92435
CW-RNN	0.127	0.88000	0.84762	0.86000	0.85375
RNN	0.156	0.87500	0.84211	0.85500	0.84850
LSTM	0.174	0.93000	0.90541	0.91800	0.91164
GRU	0.169	0.92000	0.89216	0.90500	0.89852
Transformer	0.188	0.94000	0.91089	0.92300	0.91690

**Table 6:** Time complexity of different models.

Model	Time Complexity	Formula Parameter
GWO-CW-RNN	O (T ● H ● H/M)	T represents the length of the input sequence, H represents the number of neural units in the hidden layer, and M represents the number of hidden layer modules
RNN	O (T ● K ● H)	T represents the length of the input sequence, K represents the number of output categories, and H represents the number of neural units in the hidden layer
LSTM	O (T ● D ● H)	T represents the length of the input sequence, D represents the dimension of the input feature, and H represents the number of neural units in the hidden layer
GRU	O (T ● D ● H)	T represents the length of the input sequence, D represents the dimension of the input feature, and H represents the number of neural units in the hidden layer
Transformer	O (T^2^ ● AH + TN ● D^2^)	T represents the length of the input sequence, D represents the dimension of the input feature, N represents the number of layers of the encoder and decoder, A represents the number of self-attention heads per layer, H represents the dimension of each attention head, and H = D /A

**Table 7:** Experimental results of performance comparison of optimization algorithms on the UNSW-NB15 dataset.

Algorithm	Accuracy	Precision	Recall	F1-Score
GWO-CW-RNN	0.94000	0.91234	0.92500	0.91861
PSO-CW-RNN	0.86000	0.83025	0.84500	0.83653
GA-CW-RNN	0.89000	0.86243	0.87500	0.86862
CW-RNN	0.83000	0.81225	0.81500	0.81362

**Table 8:** Experimental results of model performance comparison on the UNSW-NB15 dataset.

Model	Time per Batch (s)	Accuracy	Precision	Recall	F1-Score
GWO-CW-RNN	0.091	0.92000	0.90575	0.91000	0.90786
CW-RNN	0.132	0.83000	0.81225	0.81500	0.81362
RNN	0.148	0.82000	0.80225	0.80500	0.80361
LSTM	0.164	0.90000	0.88550	0.89000	0.88773
GRU	0.162	0.89000	0.87625	0.88000	0.87811
Transformer	0.178	0.91500	0.90225	0.90500	0.90361

**Table 9:** Experimental results of performance comparison of optimization algorithms on the NSL-KDD dataset.

Algorithm	Accuracy	Precision	Recall	F1-Score
GWO-CW-RNN	0.89000	0.88234	0.88500	0.88361
PSO-CW-RNN	0.82000	0.80225	0.80500	0.80361
GA-CW-RNN	0.85000	0.83243	0.83500	0.83362
CW-RNN	0.82000	0.81225	0.81362	0.81362

**Table 10:** Experimental results of model performance comparison on the NSL-KDD dataset.

Model	Time per Batch (s)	Accuracy	Precision	Recall	F1-Score
GWO-CW-RNN	0.089	0.90000	0.89275	0.89500	0.89386
CW-RNN	0.128	0.82000	0.81225	0.81500	0.81362
RNN	0.142	0.81000	0.80225	0.80500	0.80361
LSTM	0.159	0.89000	0.88225	0.88500	0.88361
GRU	0.158	0.88000	0.87225	0.87500	0.87361
Transformer	0.173	0.90500	0.89725	0.90000	0.89886

## Data Availability

The processed data required to reproduce these findings cannot be shared as the data also form part of an ongoing study.
